# Uncovering Novel Capsaicin Inhibitory Activity towards Human Carbonic Anhydrase Isoforms IX and XII by Combining In Silico and In Vitro Studies

**DOI:** 10.3390/antiox12051115

**Published:** 2023-05-18

**Authors:** Gianmarco Gualtieri, Annalisa Maruca, Roberta Rocca, Fabrizio Carta, Emanuela Berrino, Alessandro Salatino, Carolina Brescia, Roberta Torcasio, Manuel Crispo, Francesco Trapasso, Stefano Alcaro, Claudiu T. Supuran, Giosuè Costa

**Affiliations:** 1 Dipartimento di Scienze della Salute, Università “Magna Græcia” di Catanzaro, Viale Europa, 88100 Catanzaro, Italy; g.gualtieri@unicz.it (G.G.); salatino@unicz.it (A.S.); brescia@unicz.it (C.B.); alcaro@unicz.it (S.A.); gcosta@unicz.it (G.C.); 2 Associazione CRISEA—Centro di Ricerca e Servizi Avanzati per l’Innovazione Rurale, Località Condoleo di Belcastro, 88055 Catanzaro, Italy; 3 Net4Science S.r.l., Università “Magna Græcia” di Catanzaro, Viale Europa, 88100 Catanzaro, Italy; maruca@unicz.it; 4 Dipartimento di Medicina Clinica e Sperimentale, Università “Magna Græcia” di Catanzaro, Viale Europa, 88100 Catanzaro, Italy; roberta.torcasio@studenti.unicz.it (R.T.); manuelcrispo5@gmail.com (M.C.); trapasso@unicz.it (F.T.); 5 Dipartimento Neurofarba, Sezione di Scienze Farmaceutiche e Nutraceutiche, Università degli Studi di Firenze, Sesto Fiorentino, 50019 Florence, Italy; fabrizio.carta@unifi.it (F.C.); emanuela.berrino@uniroma1.it (E.B.); claudiu.supuran@unifi.it (C.T.S.); 6 Dipartimento di Biologia, Ecologia e Scienza della Terra (DIBEST), Università della Calabria, Arcavacata di Rende, 87036 Cosenza, Italy

**Keywords:** Capsaicin, *h*CAs, docking, anticancer

## Abstract

Hot pepper (*Capsicum annuum*) represents one of the most widespread functional foods of the Mediterranean diet, and is associated with a reduced risk of developing cardiovascular disease, cancer, and mental disorders. In particular, its bioactive spicy molecules, named Capsaicinoids, exhibit polypharmacological properties. Among them, Capsaicin (trans-8-methyl-N-vanillyl-6-nonenamide) is the most studied and reported in variegated scientific contributions for its beneficial effects, often linked to mechanisms of action unrelated to the activation of Transient Receptor Potential Vanilloid 1 (TRPV1). In this study, we present the application of in silico methods to Capsaicin for evaluating its inhibitory activity against the tumor-associated human (*h*) expressed CA IX and XII. In vitro assays confirmed Capsaicin inhibitory activity towards the most relevant tumor-related *h*CA isoforms. In particular, the *h*CAs IX and XII showed an experimental K_I_ value of 0.28 μM and 0.064 μM, respectively. Then, an A549 model of non-small cell lung cancer, typically characterized by an elevated expression of *h*CA IX and XII, was employed to test the inhibitory effects of Capsaicin in vitro under both normoxic and hypoxic conditions. Finally, the migration assay revealed that Capsaicin [10 µM] inhibits cells from moving in the A549 cells model.

## 1. Introduction

**Figure 1:**
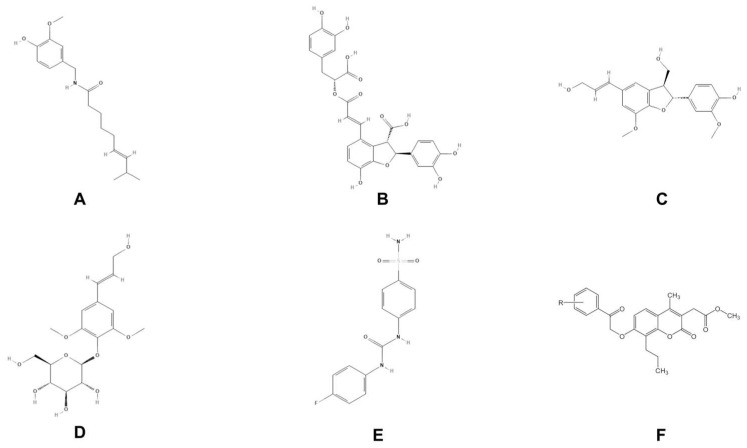
Two-dimensional structure of (**A**) Capsaicin, (**B**) Lithospermic acid, (**C**) (-)-Dehydrodiconiferyl alcohol, (**D**) Syringin, and (**E**) SLC-0111, as example of sulfonamide-based inhibitors, and (**F**) Coumarin derivative.

The traditional Mediterranean diet (MedDiet) represents a complete nutritional model widely distributed among the communities that populate the area of the Mediterranean basin [1]. This dietary pattern involves a high consumption of functional foods such as olive oil, onions, garlic, *Citrus* sp. fruits, etc. Furthermore, it involves a low intake of saturated fat sources and meat, extensive use of fibers and complex carbohydrates, a high intake of monounsaturated fatty acids, and an equal balance between omega-6 and omega-3 polyunsaturated fatty acids. Thanks to the positive impact that the MedDiet has had on human health, in 2010 United Nations Educational, Scientific and Cultural Organization (UNESCO) recognized this ancient way of living as an Intangible Cultural Heritage of Humanity [2]. Indeed, numerous scientific studies have demonstrated a close correlation between high adherence to the MedDiet and a reduced occurrence of cardiovascular, neurodegenerative, and cancer diseases [3,4]. In this scenario, thanks to its culinary art and traditional dishes, Calabria has made a huge contribution to the development and success of the MedDiet. In particular, although the poor rural area of Nicotera was selected as the reference MedDiet for Italy, the Nicotera residents, unfortunately, did not participate in Ancel Keys’ *Seven Countries Study* due to shortages of money, similar to rural areas of Greece [5]. Among the typical foods of the Mediterranean area, a prominent role is played by the hot pepper, the most emblematic spice of Calabria. Hot pepper represents the dried fruit of a plant named *Capsicum annuum*, belonging to the Solanaceae family, and it is known worldwide for its characteristic strong and spicy taste. This fruit has a considerable amount of Capsaicinoids, carotenoids, vitamins (B2, A, and C), malonic acid, citric acid, volatile oil, and a fixed one [6]. Capsaicinoids belong to the class of alkaloids with phenylalkylamine structure, and they are primarily responsible for the typical spiciness of hot pepper fruit [7]. Among all, Capsaicin is the main pungent ingredient (Figure 1A). In 1997, the burning sensation caused by Capsaicin was found to be due to the selective activation of transient receptor potential vanilloid 1 (TRPV1), a non-selective cation channel on sensory neurons that conveys information about noxious stimuli to the central nervous system [8]. Although analgesic Capsaicin’s clinical use in neuropathic pain is related to the so-called “defunctionalization” of nociceptor fibers, explained through TRPV1-mediated actions [9], it has been demonstrated that Capsaicin exerts different TRPV1-independent pharmacological effects, such as anti-inflammatory [10], bacteriostatic [11], anti-obesity [12], and antioxidant effects [13]. In particular, its antioxidant effects have been extensively studied since oxidative stress is a critical phenomenon involved with the onset of different pathologies, such as neurological disorders and cancer. Thus, Capsaicin can inhibit lipid peroxidation, prevent redox cycling of iron, enhance glutathione peroxidase and glutathione S-transferase activity, and reduce radiation-induced oxidative damages in essential endogenous antioxidant enzymes and compounds such as glutathione [14]. Its antioxidant properties are strictly related to specific groups present in the chemical structure. Both the OH phenolic group and the allylic sites are responsible for the peroxyl scavenging activity and the reaction with ^•^OCH_3_, respectively [13]. Moreover, Capsaicin has been extensively studied for anti-cancer properties, associated with its ability to interfere with multiple tumor signaling pathways and genes. Specifically, data from the literature have reported that Capsaicin provokes mutant p53 degradation in human lung cancer cell lines through autophagy. It also induces G0/G1cell-cycle arrest and apoptosis mediated by the reduced expression of human epidermal growth factor receptor, HER2, cyclin D1, and activated extra-cellular-regulated kinase in breast cancer cell lines. Furthermore, Capsaicin can trigger programmed cell death in colorectal cancers by generating reactive oxygen species, disrupting the mitochondrial transmembrane potential, and via caspase 3-mediated processes. In cholangiocarcinoma, its anti-tumor effects are linked to the suppression of cell migration and invasion through the inactivation of the Hedgehog pathway or via the AMPK-NF-kB signaling pathway. Finally, Capsaicin shows significant anti-metastatic activities with no evidence of toxicity in transgenic adenocarcinoma of the mouse prostate model and anti-angiogenesis effects via down-regulation of vascular endothelial growth factor [15]. All these scientific outcomes support the hypothesis that Capsaicin may halt the growth and division of cancer cells with many mechanisms. It is interesting to note that some scientific evidence has reported the anticancer properties of Capsaicin in cancer cell lines known to overexpress the tumor related Carbonic Anhydrase enzymes [16], although no evidence confirming its inhibitory activity against these enzymes was given. Carbonic Anhydrases (CAs, EC 4.2.1.1) are a class of metalloenzymes that catalyze the reversible reaction of the hydration of carbon dioxide (CO_2_) to bicarbonate (HCO_3_^−^) and protons (H^+^) ions. They have a crucial pathophysiological role in various human diseases, e.g., glaucoma, hemolytic anemia, osteoporosis, neurological disorders, and particularly in hypoxic cancers. To date, eight different families of CAs have been discovered (α-CAs, β-CAs, γ-CAs, δ-CAs, ζ-CAs, θ-CAs,η-Cas, and ι-CAs) [17]. According to catalytic activity, tissue distribution, and subcellular localization, human (*h*) CAs belonging to the α-family are present in 15 different isoforms. Specifically, *h*CA IX and *h*CA XII, the so-called “tumor associate isoenzymes”, fall within the membrane-bound *h*CAs [18]. Both isoforms have been validated as markers of cancer proliferation and invasion in many hypoxic tumors, and their targeted inhibition seems to impair the pH of the tumor microenvironment, with a significant reduction in malignant cells’ progression and tendency to metastasize [19]. Although sulfonamide-based *h*CA IX and *h*CA XII inhibitors showed the best results as anticancer agents [20], several natural compounds have also demonstrated significant *h*CA IX and *h*CA XII inhibitory activity, such as molecules with phenol moiety (lithospermic acid, (-)-dehydrodiconiferyl alcohol and syringin), or coumarins and psoralens analogs [17,21] (Figure 1).

In light of all this information, we explored the Capsaicin inhibition enzymatic profile regarding *h*CA IX and *h*CA XII by applying a combination of a molecular recognition approach and experimental techniques. In this context, computational methods were really helpful for the reduction of research costs and time and to speed up the drug discovery process. In particular, performing in silico studies to identify novel natural bioactive compounds with multi-target activities represents an ideal avenue to explore [22]. Thus, in this work, we performed an Induced Fit Docking protocol (IFD) in order to predict and investigate the theoretical binding affinity of Capsaicin towards *h*CA IX and *h*CA XII, taking into account the conformational changes that occur in the receptor pocket by the bound ligand. Then, thermodynamic evaluation and Molecular Dynamics simulations (MDs) were carried out to deeply analyze Capsaicin target recognition and to assess the binding stability of both generated complexes. Finally, baseline biological assays supported our computational findings, providing a rational validation of Capsaicin inhibitory activity on an in vitro cellular cancer model.

## 2. Materials and Methods

### 2.1. Molecular Modeling Studies

Concerning the molecular modeling studies, we used the three-dimensional structure of the *h*CA IX in complex with 5-(1-naphthalen-1-yl-1,2,3-triazol-4-yl)thiophene-2-sulfonamide and the crystal structure of the *h*CA XII with 2,3,5,6-tetrafluoro-4-(propylthio)benzensulfonamide, deposited in the Protein Data Bank (PDB) [23] with the PDB codes 5FL4 [24] and 5MSA [25], respectively. The receptor structures were prepared and energy-optimized by means of the Protein Preparation Wizard tool implemented in Maestro, using OPLS_2005 as the force field [26]. In particular, residual crystallographic buffer components and water molecules were removed; missing side chains were built using the Prime module, hydrogen atoms were added, and side chains’ protonation states were assigned at pH 7.4 [27,28]. Capsaicin and all ligands of the active set were prepared by means of the LigPrep Tool [29]; hydrogens were added, salts were removed, ionization states were calculated using Ionizer at pH 7.4, and then all compounds were energy minimized using OPLS_2005 as the force field.

To investigate the potential inhibitory activity of Capsaicin against *h*CAs IX and XII, we performed the IFD protocol by using the Standard Protocol and generating 20 poses for ligands. The co-crystallized ligand of each PDB template was used to set the center of the docking grid box with an automatically defined position and size based on the selected ligand. This preliminary docking was typically performed with both the receptor and the ligand “softened” by van der Waals radii scaling. By default, the scaling factor was set to 0.50 for both receptor and ligand, while residues within 5.0 Å of ligand poses were refined using Prime [30]. Dock poses were clustered using the clustering of conformer’s script in Schrödinger 2018 suite, with the aim to evaluate all possible binding modes for Capsaicin in *h*CAs pockets. Molecular Mechanics/Generalized Born Surface Area (MMGBSA) calculations were performed on the clustered poses obtained from the IFD protocol to compute the binding free energy (ΔG_bind_) of the Capsaicin complexed to both *h*CA IX and XII [31]. To validate the consistency of our molecular recognition protocol, we performed the same computational calculations on already approved and investigational *h*CA isoform IX (**2**–**5**) and XII (**1**–**5**) inhibitors, e.g., acetazolamide, zonisamide, ellagic acid, hydroflumethiazide, and benzthiazide. Thus, we obtained a *consensus* value to be applied as a filter, calculated as the average value of ΔG_bind_ of the active compounds (Table S1). Finally, the *h*CAs IX and XII, both in the apo form and complexed with the Capsaicin, were subjected to 200 ns of MDs through Desmond ver. 4.2, intending to investigate the induced-fit process of *h*CA IX and XII during the time [32]. The structures were solvated in an orthorhombic box with a buffer of 10 Å TIP3P [33] (Transferable Intermolecular Potential 3-Point) water, and Na^+^ ions were added to neutralize the system charge. The MDs were performed using the following conditions: isothermal–isobaric (NPT) ensemble, a temperature of 300 K, and a pressure of 1 bar.

### 2.2. Inhibition Assay

An Applied Photophysics stopped-flow instrument evaluated the CA-catalyzed carbon dioxide hydration. A 0.2 mM quantity of phenol red was selected as the indicator, at an absorbance maximum of 557 nm, with 10 mM Hepes (pH 7.5) as buffer and 0.1 M Na_2_SO_4_ (for maintaining constant ionic strength), followed by the reaction for 10–100 s. The substrate concentrations ranged from 1.7 to 17 mM. Then, 10 mM stock solutions of each inhibitor were made in deionized water with 10% of dimethylsulfoxide (DMSO), and dilutions up to 0.001 μM were performed with the assay buffer. Both compounds and enzyme were preincubated for 6 h (standard assay at room temperature (i.e., r.t.) for the formation of the E–I complex. The K_I_s were extrapolated by non-linear least squares methods using PRISM 3 and the Cheng–Prusoff equation and were reported as the mean from at least three different determinations. Enzyme concentrations in the assay system were in the range of 5–12 nM. All enzymes were recombinant [34,35].

### 2.3. Cell Cultures

For the in vitro analysis, the human non-small cell lung cancer A549 cell line [36] was chosen as *h*CAIX^+^ and *h*CAXII^+^ preclinical models. Cells were plated in RPMI 1640 (Life Technologies Corp., Carlsbad, CA, USA) culture medium, enriched with 10% Fetal Bovine Serum and 1% Pen/Strep. The cultures were incubated at 37 °C either in normoxic or in hypoxic conditions, being cells cultured in 7% O_2_ and 5% CO_2_ or in 1% O_2_ in a dedicated Eppendorf Galaxy 48R incubator, respectively.

### 2.4. Growth Curve Assay

Cell growth was evaluated by implanting 105 A549 cells in 35 mm Petri dishes and, after 24 h starvation with serum free medium, treating with [0.1–1–10–100 µM] of Capsaicin (SIGMA, Cod.1003230823). Plates were then incubated under normoxic conditions for 24 h, 48 h, and 72 h and at each time point cells were tripsinized and counted in Burker’s chamber, using the Trypan Blue dye for the discrimination of dead cells.

### 2.5. MTT Viability Test

To test cell viability, a 3-(4,5-dimethylthiazol-2-yl)-2,5-diphenyl-2H-tetrazolium bromide (MTT) assay was performed 48 h and 72 h after Capsaicin [10 µM] treatment. An MTT assay carried out according to Costa et al.’s protocol [17]. Treatment with Capsaicin [10 µM] was made in parallel with [10 µM] inhibitors of *h*CA IX and XII, including SLC-0111, CF3-SLC, and FL88. All substances used in this study were dissolved in DMSO. Treated cells were incubated in both normoxic and hypoxic conditions.

### 2.6. Wound Healing Assay

The effects of Capsaicin [10 µM] on A549 cells migration were determined as previously described by Salatino et al. [37]. A549 cells were seeded into P60 culture dishes with complete RPMI-1640 medium. When cells reached 90% confluence, a wound was manually created through the scraping of the cell monolayer using a sterile p200 tip. After that, the culture medium was changed and cells were kept in culture both in the presence and in the absence of Capsaicin [10 µM] for up to 24 h, 48 h, and 72 h after the scratch.

To quantify the wound closure over time, images of the wound area were captured using a digital inverted microscope EVOS XL (life technologies) and data of area were processed using ImageJ software ver 32 bit (National Institutes of Health, Bethesda, MD, USA).

### 2.7. Western Blot Analysis

Total protein extracts were prepared through RIPA buffer methods, using the standard protocol described by Salatino et al. [36]. To separate each protein sample (30 μg) we uploaded them on 10% SDS-PAGE and then transferred them to nitrocellulose membranes. Then, membranes were incubated with primary antibody anti-MMP-9 (1:500.*-*Cod.: sc-21733*-*Santa Cruz Biotechnology, Dallas, TX, USA) at 4 °C overnight. Immunoreactive bands were detected using the ECL Western blotting detection system (Santa Cruz Biotechnology, Dallas, TX, USA). To normalize the protein samples loaded, we used anti-tubulin antibody (1:1000*-*Cod.: 2144 Cell Signaling). Densitometry analysis was performed using ImageJ software.

### 2.8. Statistical Analysis

All tests were performed in triplicate and results were expressed as mean ± standard deviations (SD). Differences between groups were analyzed using the two-way test (ANOVA) followed by the Bonferroni test. The analysis was conducted using the GraphPad Prism Software ver 8.0.2. (San Diego, CA, USA) and the differences were considered significant at ** *p* < 0.01 and *** *p* < 0.001.

## 3. Results

### 3.1. Computational Prediction of Capsaicin Binding Affinity against hCA IX and hCA XII

Firstly, we evaluated the Capsaicins’ free energy of binding (ΔG_bind_) towards both *h*CAs’ active sites. To predict if the affinity of the Capsaicin allowed good interaction with the selected targets, we considered as a cut-off the consensus values of −55.66 and −58.81 Kcal/mol for *h*CA IX and XII, respectively (Table S1). For Capsaicin, we obtained three different binding modes within both *h*CAs pockets, and all exhibited ΔG_bind_ values better than the cut-off obtained from the active set. In particular, the ΔG_bind_ was between −96.92 and −101.91 Kcal/mol for *h*CA IX, while it ranged between −64.37 and −94.92 Kcal/mol for *h*CA IX (Table 1). Thus, we proposed this natural compound as a new inhibitor of both *h*CAs isoforms.

The ΔG_bind_ energy components (ΔG_coul_, ΔG_lipo_, ΔG_solvGB_, ΔG_vdW_) are reported and analyzed in the Supplementary Materials (Section “Thermodynamic Analysis”, Tables S2 and S3).

**Figure 2:**
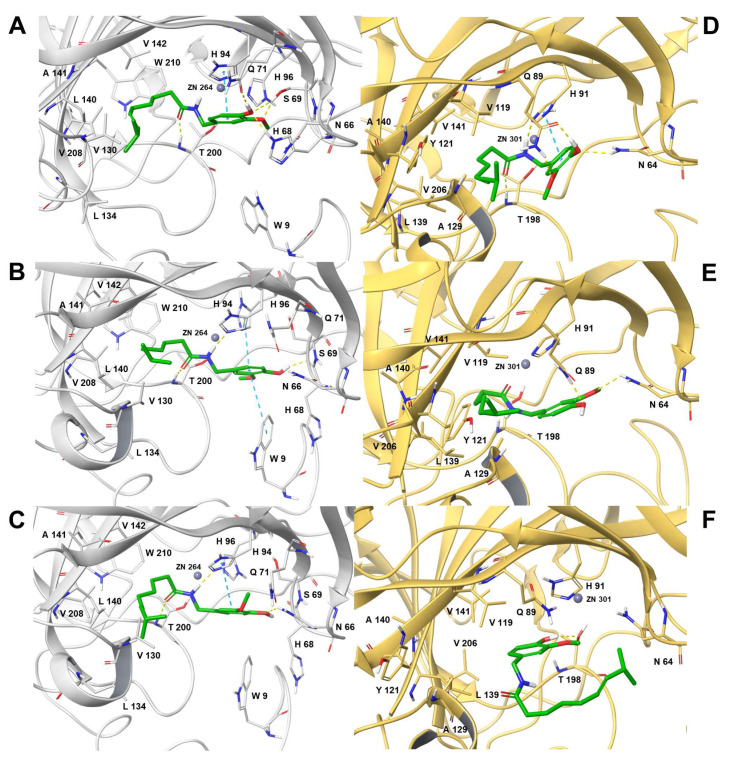
Three-dimensional representation of the Capsaicin complexed with (**A**–**C**) *h*CA IX and (**D**–**F**) *h*CA XII. The ligands are depicted as green sticks, the zinc ion as a grey sphere and the amino acid residues involved in the most relevant contacts with ligands as gray and yellow sticks. Conversely, *h*CA IX and *h*CA XII are depicted as grey and yellow cartoons, respectively. H-bonds and stacking interactions are shown as yellow and cyan dash lines, respectively.

By evaluating the docking poses, Capsaicin is well recognized in the catalytic binding site of both *h*CA IX and XII, as shown in Figure 2.

For the *h*CA IX, the three main binding modes displayed the same arrangement of Capsaicin in the binding site. In particular, the alkyl chain was well enclosed in the hydrophobic pocket formed by the residues L134, V130, L140, A141, V142, V208, and W210. The amide group faced the zinc ion by establishing electrostatic interactions with it and an acceptor Hydrogen bond (H-bond) between its carbonyl group and the backbone of T200. Moreover, in binding modes 2 and 3, we observed that the amide group was also involved in a donor H-bond with the side chain of H94. In all three binding modes, the methoxy-phenol moiety involved a stacking interaction with the side chain of H94, even if it was directed to a hydrophilic cavity with distinct orientations and the following establishment of different electrostatic contacts (Figure 2A–C). Specifically, in binding mode 1, the methoxy moiety was involved in an acceptor H bond with the side chain of S69. Conversely, the phenol group accepted two H-bonds from the side chain of S69 and H68 and acted as a donor towards the side chain of Q71 (Figure 2A). Regarding binding mode 2, the benzene ring and the phenolic group were involved in a stacking interaction with the side chain of W9 and a donor H-bond with the backbone of N66, respectively (Figure 2B). Conversely, in binding mode 3, the phenolic group established an acceptor H-bond with the side chain of N66 (Figure 2C).

For the pocket of isoform XII, the docking poses of Capsaicin (Figure 2D–F) showed higher variability than the complexes of *h*CA IX (Figure 2A–C). In particular, binding mode 3 (Figure 2E) showed the methoxy and phenolic groups facing the zinc to favor its coordination. Moreover, the phenolic moiety established an H-bond with the T198 side chain. This pose is generally characteristic of phenolic compounds, even if, for the Capsaicin, it seemed energetically unfavored due to the reduced hydrophobic interactions between ligand and receptor, as can also be seen from the energetic components (Table S2). Thus, this observation can be related to the greater exposure of the alkyl chain to the solvent and its orientation towards hydrophilic residues, such as N64, H66, and S67. An arrangement similar to that obtained for *h*CA IX was observed in the other two binding modes of Capsaicin with *h*CA XII. In particular, the alkenyl chain establishes several hydrophobic interactions with the residues A129, Y121, V119, L139, A140, V141, and V206, while the amide moiety faces the zinc to form electrostatic interactions (Figure 2D,E). In binding mode 1, the amide moiety is also involved in two H-bonds with the backbone of T198 and the side chain of H91 (Figure 2D). The main differences between binding modes 1 and 2 were related to the methoxy–phenolic portion, which showed a flipped conformation in the two docking poses (Figure 2D,E). Specifically, in binding mode 1 (Figure 2D), we observed a stacking interaction between the benzene ring of the ligand and the side chain of H91, which was absent in binding mode 2. Moreover, in binding mode 2 (Figure 2E), two acceptor H-bonds were established between the phenolic and methoxy groups with the side chains of Q89 and N64, respectively. Conversely, in binding mode 1, the two acceptor H-bonds were inverted, and a donor H-bond was also observed between the phenolic group and the side chain of Q89.

**Figure 3:**
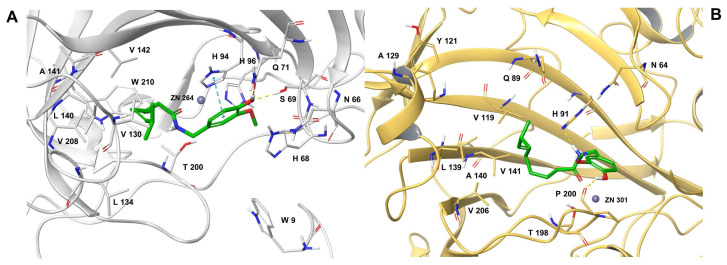
Three-dimensional representation of the most populated cluster of Capsaicin complexed with (**A**) *h*CA IX and (**B**) *h*CA XII. The ligands are depicted as green sticks, the zinc ion as a grey sphere, and the amino acid residues involved in the most relevant contacts with ligands as gray and yellow sticks. Conversely, *h*CA IX and *h*CA XII are depicted as grey and yellow cartoons, respectively. H-bonds and stacking interactions are shown as yellow and cyan dash lines, respectively.

Finally, we investigated the binding stability of the best thermodynamic complexes of Capsaicin to the *h*CA isoforms IX and XII. For the complex of the Capsaicin with the IX isoform of *h*CA, we observed a higher stability of the complexed protein than the *apo* form (Figure S1A) by calculating RMSD values on the Cα of the protein. Thus, Capsaicin keeps the protein structure more stable, preventing the achievement of RMSD values higher than 2 Å, with an average RMSD value of 1.4 Å, highlighting the lack of relevant conformational changes during the MDs. Conversely, the receptor in the *apo* form showed a less stable RMSD value trend, with an average value of 2.07 Å, reaching peaks of 2.5 Å. Additionally, for the *h*CA XII-Capsaicin complex, MDs showed almost-stable behavior of the protein during the 200 ns simulation (Figure S1B), as observed for isoform IX. In particular, the average RMSD value calculated on the Cα of the protein complexed with Capsaicin is 1.43 Å, corresponding to that obtained for the isoform IX. Conversely, *h*CA XII in the *apo* form showed a less stable RMSD trend, with a mean value of 1.68 Å and peaks of 2.3 Å, after the first 100 ns of simulation. On the other hand, after an early adjustment, the RMSD trend of Capsaicin identified a stable binding mode in both pockets (Figure S2). Thus, all the structures sampled during the MDs were clusterized, and for both isoforms the most populated complex was analyzed (Figure 3). For isoform IX, we observed the conservation of most of the interactions observed in docking (Figure 2A), except for two acceptor H-bonds involving the phenolic and the carbonyl groups with the side chain of H68 and the backbone of T200 (Figure 3A). In particular, the loss of the H-bond with the T200 was due to the flip of the amide moiety, which allowed the orientation of the carbonyl group towards the zinc for establishing electrostatic interactions. Conversely, in the *h*CA XII binding pocket we observed the conservation of the donor H-bond between the amidic moiety and the side chain of H91, together with the several hydrophobic interactions involving the alkenyl chain of Capsaicin and A129, Y121, V119, L139, A140, V141, and V206 (Figure 3B). Thus, compared to the docking pose (Figure 2D), the Capsaicin modified the position of the methoxyphenyl ring with the formation of a donor H-bond between the phenol and the carbonyl group of P200 (Figure 3B).

### 3.2. Carbonic Anhydrase Inhibition Assay

Capsaicin was investigated in vitro for its ability to inhibit the most physiologically relevant *h*CA isoforms, I, II, VA, VB, IX, and XII, by means of the stopped-flow technique. The obtained data are reported in Table 2 as K_I_ values and compared to the standard CA inhibitor of the sulfonamide type acetazolamide (AAZ).

As reported in Table 2, Capsaicin was an ineffective inhibitor of the *h*CA I and II isoforms (i.e., K_I_s > 100 μM), whereas it showed submicromolar values for the remaining isozymes considered. Specifically, Capsaicin as a ligand was 1.7-fold less potent for the constitutively expressed mitochondrial *h*CAs VA over the VB, with K_I_ values of 0.75 and 0.44 μM, respectively. Quite interesting, for the purposes of the research herein reported, were the inhibition potencies obtained for the tumor-related isoforms. The *h*CAs IX showed a K_I_ value of 0.28 μM, thus being 10.8-fold less effective when compared to the reference and prototypic CA inhibitor AAZ. It is noteworthy that Capsaicin resulted in a 4.4-fold more effective ligand for the *h*CA XII isoform. The in vitro kinetic assay reported an experimental K_I_ value of 0.064 μM, which was the lowest among all the enzyme panels reported. In analogy to the *h*CA IX, a similar trend was observed when the Capsaicin *h*CA XII values were compared to AAZ, the latter being 11.2-fold more potent.

### 3.3. Capsaicin Inhibits A549 Cell Growth Similarly to hCA IX and XII Inhibitors

**Figure 4:**
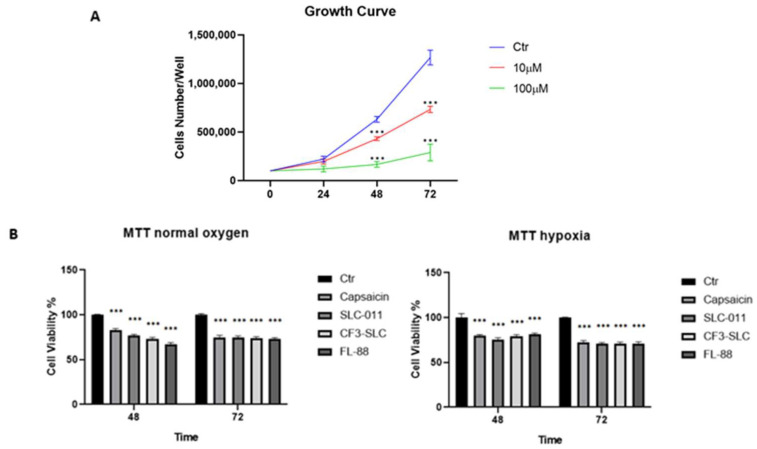
The pictures show the in vitro inhibitory effects of capsaicin in the A549 cell model: (**A**) dose-dependent growth curve of A549 cells treated with different concentrations of Capsaicin 0.1 μM (not shown), 1 μM (not shown), 10 μM and 100 μM for 24 h, 48 h and 72 h. Cells were counted in Burker’s chamber, using the Trypan Blue. (**B**) MTT assay was performed to determine cell viability and cytotoxicity after 48 h and 72 h treatment with Capsaicin [10 µM], in parallel with SLC-0111, CF3-SLC, and FL-88 [10 µM], well-known *h*CA XI and *h*CA XII inhibitors. *** *p* < 0.001.

To choose a better suitable dose for our experiment and when aiming to have better growth cell inhibition limiting cytotoxicity, a growth curve assay was performed exposing A549 at an increasing range of Capsaicin concentrations (from [0,1] to [100] µM) for 24 h, 48 h, and 72 h, comparing them with the untreated control. As shown in Figure 4A, reduced cell growth could be observed as early as 24 h, when samples were exposed to the maximum tested dose of Capsaicin [100 µM]. With increased temporal exposure, respectively, at 48 h and 72 h, we obtained a significant inhibitory effect even at a lower dose of [10 µM]. This reasonably cell-tolerated dose gave us the possibility of comparing the effects of Capsaicin with SLC-0111, CF3-SLC, and FL88, well-known *h*CA XI an *h*CA XII inhibitor substances. Therefore, the viability assay was conducted on A459 cells treated with Capsaicin [10 µM] under both normoxic and hypoxic conditions. These different conditions of incubation were helpful to obtain enhanced expressions of *h*CA IX and *h*CA XII in our cellular model, as described by Ilie et al. [38]. As reported in Figure 4B, Capsaicin affects A549 viability greater under hypoxic than normoxic conditions at both 48 h and 72 h of treatment. Better significance was evaluated at 72 h (~30% cells viability reduction) compared to the untreated control. In addition, the progressive decrease in viability under hypoxia caused by capsaicin was significantly appreciable at 48 h, showing a trend comparable to *h*CA inhibitors SLC-0111, CF3-SLC, and FL88, presumably due to a higher expression of *h*CA IX and *h*CA XII under hypoxic conditions.

### 3.4. Capsaicin [10 µM] Suppresses A549 Cell Migration Targeting Matrix Metallo-Peptidase 9

**Figure 5:**
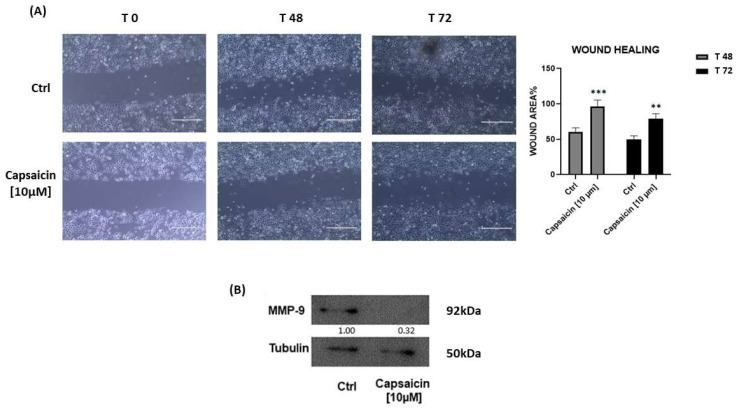
(**A**) Optical microscopy images of wound healing assay on A549 cells, untreated vs. Capsaicin [10 µM] samples acquired at different time points of 0, 24 h (not shown), 48 h, and 72 h. (**B**) Representative Western Blot of MMP-9 from protein extracted by A459 cells culture. β-Tubulin was used for normalization control. In both conducted experiments, densitometry analysis was elaborated using ImageJ Software. ** *p* < 0.01 and *** *p* < 0.001.

Performing a wound healing test, in order to understand if Capsaicin could affect A549 migration, we observed the effects of [10 µm] treatment on injured A459 cells. The efficient shift from the borders of the scratch to wound healing was low in the sample treated with Capsaicin [10 µM] within 24 h (data not shown) and increased over time both at 48 h and 72 h where the wound area was 40% greater, as shown in Figure 5.

In addition, we conducted a Western blot analysis on total proteins extracted from the above-mentioned cultured cells, with the purpose of understanding if the treatment with Capsaicin [10 µM] effectively acts on cellular motility.

According to Figure 4B, the downregulation of Matrix Metallo-Peptidase 9 (MMP-9) resulted from the A549 Capsaicin-treated sample, supporting the hypothesis of an inhibitory Capsaicin effect on A549 cell migration.

## 4. Discussion

Recent studies have described a key role of *h*CA IX and *h*CA XII in the pathogenesis of different cancer types [39], paving the idea for the study of these enzymatic isoforms as a potential target for the development of new cancer therapies. The two isoforms IX and XII of *h*CA are overexpressed in many tumors and correlate with a hypoxic and acidified microenvironment that plays an important role in tumor progression and resistance to therapy [40,41]. In addition to this, biochemical studies have already described *h*CA I and II as a potential inhibitor target of Capsaicin [42], a potent nutraceutical compound, with an anticancer activity previously reported [43]. In light of this, we performed computational studies to assess the potential inhibitory activity of Capsaicin towards the isoforms IX and XII of *h*CA. The thermodynamic analysis proved a suitable affinity of the natural compound with both *h*CA IX and XII by highlighting the lipophilic (ΔG_lipo_) and Van der Waals (ΔG_vdW_) contributions as the most important for Capsaicin binding. Indeed, the analysis of the principal clusters for the binding modes obtained from the IF protocol revealed the good fit of the alkenyl chain into a hydrophobic pocket of both enzymes as the driving force for the docking of Capsaicin. Confirming the computational prediction, in vitro assays on the panel of the physiologically most relevant *h*CA isoforms pointed out the lowest experimental K_I_ value for the IX and XII. Based both on results obtained from computational and in vitro analysis and investigations of the reported literature, we decided to test the inhibitory effects of Capsaicin in vitro, in an A549 model of non-small cell lung cancer. An elevated expression of *h*CA IX and XII was proved from other research groups in tumor forms of non-small cell lung cancer [44,45].

The use of this cellular model, previously described as *h*CAIX^+^ and *h*CA XII^+^ [43,44], together with the use as a control of known inhibitory agents that target CAIX and CA XII, enabled us to achieve an indirect proof of principle of the *h*CA IX/XII-Capsaicin inhibition.

From our results, the inhibitory action of Capsaicin on the A549 cells viability was found to have a parallel course to the compounds SLC-0111, CF3-SLC, and FL88 after 72 h of treatment. Moreover, incubating cell cultures under hypoxia conditions showed a parallel effect early, after 48 h of treatment. The comparison between inhibitors of *h*CA IX and XII and Capsaicin, on the same appropriate cellular model, supported the idea that *h*CA could be a molecular target of Capsaicin.

In addition, another proof was obtained through the migration assay, where Capsaicin [10 µM] inhibited cells moving in the A549 cells model. In agreement with our supposition, other groups had previously demonstrated that an inhibitory effect on the migration of further cancer cells was linked to the suppression of *h*CA IX e XII [46,47].

Indeed, the results obtained from our Western blot analysis support what has been said so far. Yang, J.S. et al. described how *h*CA IX induces the expression of MMP-9. As proof of this, we demonstrated, for the first time, how MMP-9 is down-regulated after Capsaicin treatment, identifying an additional element that may support the idea that capsaicin acts on *h*CA IX.

Although in vitro results are nothing more than a statement of principle, additional investigations will be required to characterize the molecular response of CA IX and XII to Capsaicin treatment. However, our results, achieved from the in silico test, together with the in vitro assay and the knowledge present in literature, suggest a potential role of *h*CA IX and XII as a target of Capsaicin.

## Figures and Tables

**Table 1:** ΔG_bind_ values for the complexes of *h*CA IX and *h*CA XII with Capsaicin. The reported values are expressed in Kcal/mol.

ΔG_bind_ (Kcal/mol)	*h*CA IX	*h*CA XII
Binding mode 1	−101.91	−94.92
Binding mode 2	−98.78	−83.81
Binding mode 3	−96.92	−64.37

**Table 2:** Inhibition data of *h*CA isoforms I, II, VA, VB, IX, and XII with Capsaicin and the standard sulfonamide inhibitor acetazolamide (AAZ) by the stopped-flow CO_2_ hydrase assay [38].

K_I_ (µM) *						
	*h*CA I	*h*CA II	*h*CA VA	*h*CA VB	*h*CA IX	*h*CA XII
Capsaicin	>100	>100	0.75	0.44	0.28	0.064
AAZ	0.250	0.012	0.063	0.054	0.026	0.0057

* Mean from assays carried out in triplicate (errors were in the range of ±5–10% of the reported values).

## Data Availability

Data are contained in the article and Supplementary Materials.

## Supplementary Materials

The following supporting information can be downloaded at: https://www.mdpi.com/article/10.3390/antiox12051115/s1, Table S1: Two-dimensional structures and binding free energy values (ΔG_bind_) for each already-approved inhibitor of both *h*CA IX and XII isoforms. Additionally, average ΔG_bind_ values for both isoforms are shown. All reported values are expressed in Kcal/mol; Thermodynamic analysis; Table S2: Values of ΔG_bind_ energy components (ΔG_coul_, ΔG_lipo_, ΔG_solvGB_, ΔG_vdW_) for the complexes of *h*CA IX and *h*CA XII with Capsaicin. The reported values are expressed in Kcal/mol; Table S3: Values of the ΔG_bind_ energy components (ΔG_coul_, ΔG_lipo_, ΔG_solvGB_, ΔG_vdW_) related to the complexes of *h*CA IX and *h*CA XII with the known active compounds. The reported values are expressed in Kcal/mol; Figure S1: Trend of the RMSD value calculated on the Cα of (A) *h*CA IX and (B) *h*CA XII in the apo form (black line) and in complex with the Capsaicin (red line). The RMSD values are reported in Å; Figure S2: RMSD trend of Capsaicin complexed with (A) the *h*CA IX and (B) the *h*CA XII, during MDs. The RMSD values, reported in Å, are calculated on the heavy atoms of the ligand after superimposition on the protein.
