# Supplementary material for: Uncovering Novel Capsaicin Inhibitory Activity towards Human Carbonic Anhydrase Isoforms IX and XII by Combining In Silico and In Vitro Studies
Source: Antioxidants (Basel). 2023 May 18;12(5):1115. doi: 10.3390/antiox12051115 (PMC10215620; doi:10.3390/antiox12051115)
Supplement: Supplementary file 1 [file antioxidants-12-01115-s001.zip › antioxidants-2374181-supplementary.pdf]

antioxidants

MDPI

Supplementary Material

Uncovering Novel Capsaicin Inhibitory Activity towards Human Carbonic Anhydrase Isoforms IX and XII by Combining In Silico and In Vitro Studies

Gianmarco Gualtieri [1,2], Annalisa Maruca [3], Roberta Rocca [2,3,4,*], Fabrizio Carta [5], Emanuela Berrino [5], Alessandro Salatino [1], Carolina Brescia [1], Roberta Torcasio [4,6], Manuel Crispo [4], Francesco Trapasso [4], Stefano Alcaro [1,2,3], Claudiu T. Supuran [5] and Giosuè Costa [1,3]

[1] Dipartimento di Scienze della Salute, Università "Magna Græcia" di Catanzaro, Viale Europa, 88100 Catanzaro, Italy; g.gualtieri@unicz.it (G.G.); salatino@unicz.it (A.S.); brescia@unicz.it (C.B.); alcaro@unicz.it (S.A.); gcosta@unicz.it (G.C.)

[2] Associazione CRISEA—Centro di Ricerca e Servizi Avanzati per l'Innovazione Rurale, Località Condoleo di Belcastro, 88055 Catanzaro, Italy

[3] Net4Science Srl, Università "Magna Græcia" di Catanzaro, Viale Europa, 88100 Catanzaro, Italy; maruca@unicz.it

[4] Dipartimento di Medicina Clinica e Sperimentale, Università "Magna Græcia" di Catanzaro, Viale Europa, 88100 Catanzaro, Italy; roberta.torcasio@studenti.unicz.it (R.T.); manuelcrispo5@gmail.com (M.C.); trapasso@unicz.it (F.T.)

[5] Dipartimento Neurofarba, Sezione di Scienze Farmaceutiche e Nutraceutiche, Università degli Studi di Firenze, Sesto Fiorentino, 50019 Florence, Italy; fabrizio.carta@unifi.it (F.C.); emanuela.berrino@uniroma1.it (E.B.); claudiu.supuran@unifi.it (C.T.S.)

[6] Dipartimento di Biologia, Ecologia e Scienza della Terra (DIBEST), Università della Calabria, Arcavacata di Rende, 87036 Cosenza, Italy

* Correspondence: rocca@unicz.it; Tel.: +39-0961-369-4198

Table of Contents

Table S1. 2D structures and binding free energy values (ΔG_{bind}) for each already approved inhibitor of both *h*CA IX and XII isoform. Also, average ΔG_{bind} values for both isoforms are shown. All reported values are expressed in Kcal/mol.

Thermodynamic analysis

Table S2. Values of ΔG_{bind} energy components (ΔG_{coul}, ΔG_{lipo}, ΔG_{solvGB}, ΔG_{vdW}) for the complexes of *h*CA IX and *h*CA XII with Capsaicin. The reported values are expressed in Kcal/mol.

Table S3. Values of the ΔG_{bind} energy components (ΔG_{coul}, ΔG_{lipo}, ΔG_{solvGB}, ΔG_{vdW}) related to the complexes of *h*CA IX and *h*CA XII with the known active compounds. The reported values are expressed in Kcal/mol.

Figure S1. Trend of the RMSD value calculated on the Cα of **A)** *h*CA IX and **B)** *h*CA XII,in the *apo* form (black line) and in complex with the capsaicin (red line). The RMSD values are reported in Å.

Figure S2. RMSD trend of capsaicin complexed with **A)** the *h*CA IX and **B)** the *h*CA XII, during MDs. The RMSD values, reported in Å, are calculated on the heavy atoms of the ligand after the superimposition on the protein.

Table S1. 2D structures and binding free energy values (*ΔG_{bind}*) for each already approved inhibitor of both *h*CA IX and XII isoform. Also, average *ΔG_{bind}* values for both isoforms are shown. All reported values are expressed in Kcal/mol.

		***h*AC IX**	***h*AC XII**
**Compound**	**2D structure**	**ΔG_{bind} (Kcal/mol)**	**ΔG_{bind} (Kcal/mol)**
Benzthiazide	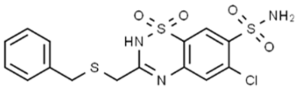	-79.85	-71.78
Ellagic acid	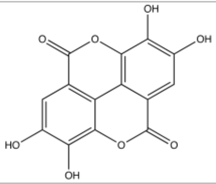	-55.96	-73.50
Zonisamide	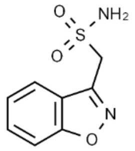	-44.01	-43.28
Hydroflumethiazide	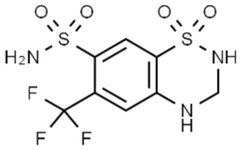	-42.83	-61.74
Acetazolamide	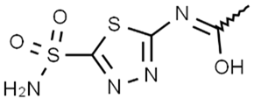	/	-43.76
**Average ΔG_{bind} (Kcal/mol)**		-55.66	-58.81

Thermodynamic analysis

To better investigate the affinity of capsaicin towards *h*CA IX and XII, we performed ΔG_{bind} energy component analysis, taking into account the active set (Table S2 and Table S3).

Table S2. Values of ΔG_{bind} energy components (ΔG_{coul}, ΔG_{lipo}, ΔG_{solvGB}, ΔG_{vdW}) for the complexes of *h*CA IX and *h*CA XII with Capsaicin. The reported values are expressed in Kcal/mol.

		**ΔG_{Coul} (Kcal/mol)**	**ΔG_{lipo} (Kcal/mol)**	**ΔG_{solvGB} (Kcal/mol)**	**ΔG_{vdW} (Kcal/mol)**
***h*CA IX**	**Binding mode 1**	-34.62	-51.65	22.12	-41.02
	**Binding mode 2**	-19.31	-67.63	32.78	-51.92
	**Binding mode 3**	-24.35	-54.98	27.61	-48.11
***h*CA XII**	**Binding mode 1**	-37.77	-47.97	35.11	-44.26
	**Binding mode 2**	-13.34	-52.91	23.96	-43.51
	**Binding mode 3**	-13.85	-34.35	20.53	-40.58

As observed for the active set, we point out both the lipophilic (ΔG_{lipo}) and Van der Waals (ΔG_{vdW}) contributions as the most important for the capsaicin binding of the *h*CA IX. Specifically, compared to the Benzthiazide, which exhibited the best hydrophobic contribution in the binding to *h*CA IX among the reference compounds, capsaicin showed

improved both ΔG_{lipo} and ΔG_{vdW}. Specifically, the best values for these energetic components were observed for the binding mode 2, with ΔG_{lipo} and ΔG_{vdW} equal to -67.63 and -51.92 Kcal/mol. Considering the best thermodynamic complex of capsaicin with the *h*CA IX, the electrostatic contribution, with a value of -34.62 Kcal/mol, appeared significant for the capsaicin binding to *h*CA IX. Conversely, in the active set, this contribution is lowly for most ligands, except for the ellagic acid, which has shown a higher ΔG_{coul} value of 7 Kcal/mol than capsaicin. Furthermore, capsaicin, similar to ellagic acid, has a higher solvation penalty than the other active compounds.

As observed for *h*CA IX, the complex of capsaicin with *h*CA XII showed improved ΔG_{lipo} and ΔG_{vdW} compared to the active set, reconfirming the hydrophobic contribution as the most important one for its binding to these targets. Also, for *h*CA XII, the best values for these energetic components were observed for binding mode 2, with ΔG_{lipo} and ΔG_{vdW} equal to -52.91 and -43.51 Kcal/mol. Regarding the electrostatic component, capsaicin exhibited the ΔG_{coul} best value of about -37.77 Kcal/mol in binding mode 2. Conversely, in the other docking poses, although the ΔG_{coul} is also higher than those exhibited by the active compounds Acetazolamide and Ellagic acid complexed with *h*CA XII, it is advantageous compared to Benzthiazide, Hydroflumethiazide, and Zonisamide, in any case. Finally, the solvation energy shared a similar value in the complexes of capsaicin with both *h*CA isoforms.

Table S3. Values of the ΔG_{bind} energy components (ΔG_{coul}, ΔG_{lipo}, ΔG_{solvGB}, ΔG_{vdW}) related to the complexes of *h*CA IX and *h*CA XII with the known active compounds. The reported values are expressed in Kcal/mol.

	**Compound**	**ΔG_{coul}** (Kcal/mol)	**ΔG_{lipo}** (Kcal/mol)	**ΔG_{solvGB}** (Kcal/mol)	**ΔG_{vdW}** (Kcal/mol)
***h*CA IX**	Benzthiazide	-3.15	-31.26	-0.29	-43.74
	Hydroflumethiazide	-2.70	-7.73	-7.08	-26.27
	Ellagic acid	-29.11	-19.38	29.01	-35.75
	Zonisamide	-6.97	-21.82	12.38	-27.16
***h*CA XII**	Benzthiazide	-8.17	-28.75	3.18	-40.51
	Hydroflumethiazide	-3.56	-16.92	-12.38	-28.69
	Ellagic acid	-23.70	-24.64	13.97	-35.72
	Zonisamide	-10.97	-19.44	14.15	-27.09
	Acetazolamide	-21.16	-10.48	14.16	-27.60

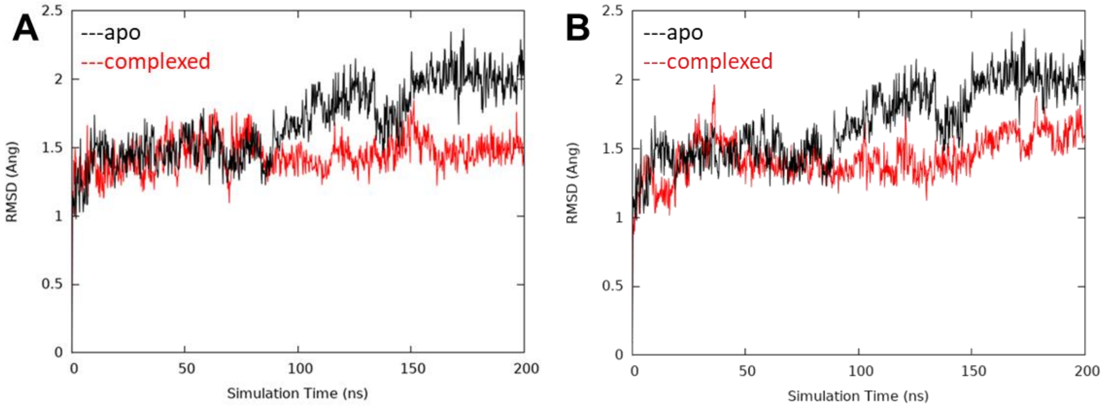

Figure S1. Trend of the RMSD value calculated on the Cα of **A)** *h*CA IX and **B)** *h*CA XII,in the *apo* form (black line) and in complex with the capsaicin (red line). The RMSD values are reported in Å.

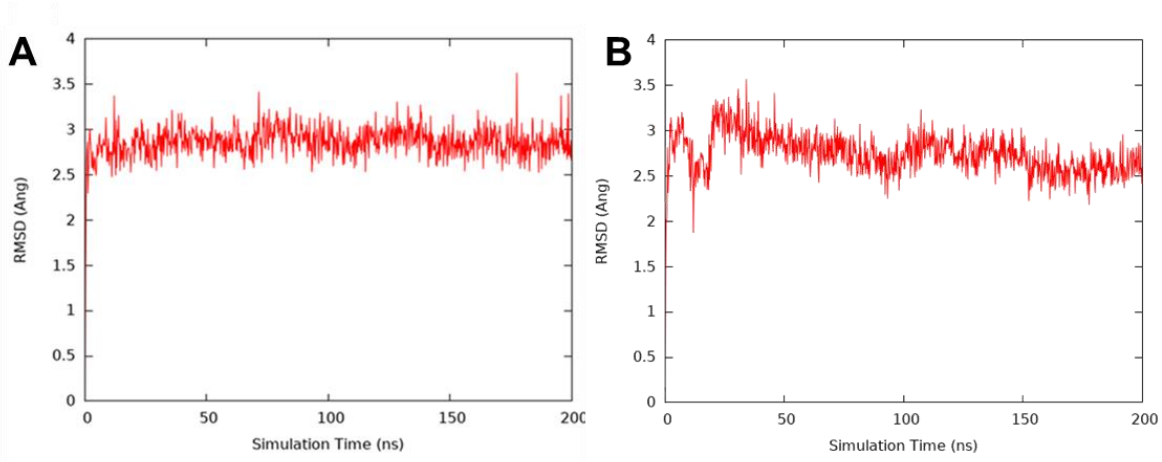

Figure S2. RMSD trend of capsaicin complexed with **A)** the *h*CA IX and **B)** the *h*CAXII, during MDs. The RMSD values, reported in Å, are calculated on the heavy atoms of the ligand after the superimposition on the protein.
